# Prion-Like Propagation of Protein Misfolding and Aggregation in Amyotrophic Lateral Sclerosis

**DOI:** 10.3389/fnmol.2019.00262

**Published:** 2019-11-01

**Authors:** Luke McAlary, Steven S. Plotkin, Justin J. Yerbury, Neil R. Cashman

**Affiliations:** ^1^Illawarra Health and Medical Research Institute, University of Wollongong, Wollongong, NSW, Australia; ^2^Molecular Horizons and School of Chemistry and Molecular Bioscience, Faculty of Science, Medicine and Health, University of Wollongong, Wollongong, NSW, Australia; ^3^Department of Physics and Astronomy, University of British Columbia, Vancouver, BC, Canada; ^4^Genome Sciences and Technology Program, University of British Columbia, Vancouver, BC, Canada; ^5^Djavad Mowafaghian Centre for Brain Health, University of British Columbia, Vancouver, BC, Canada

**Keywords:** amyotrophic lateral sclerosis, protein misfolding, protein aggregation, prion, proteostasis

## Abstract

The discovery that prion protein can misfold into a pathological conformation that encodes structural information capable of both propagation and inducing severe neuropathology has revolutionized our understanding of neurodegenerative disease. Many neurodegenerative diseases with a protein misfolding component are now classified as “prion-like” owing to the propagation of both symptoms and protein aggregation pathology in affected individuals. The neuromuscular disorder amyotrophic lateral sclerosis (ALS) is characterized by protein inclusions formed by either TAR DNA-binding protein of 43 kDa (TDP-43), Cu/Zn superoxide dismutase (SOD1), or fused in sarcoma (FUS), in both upper and lower motor neurons. Evidence from *in vitro*, cell culture, and *in vivo* studies has provided strong evidence to support the involvement of a prion-like mechanism in ALS. In this article, we review the evidence suggesting that prion-like propagation of protein aggregation is a primary pathomechanism in ALS, focusing on the key proteins and genes involved in disease (TDP-43, SOD1, FUS, and *C9orf72*). In each case, we discuss the evidence ranging from biophysical studies to *in vivo* examinations of prion-like spreading. We suggest that the idiopathic nature of ALS may stem from its prion-like nature and that elucidation of the specific propagating protein assemblies is paramount to developing effective therapies.

## Neurodegenerative Disease: A Common Pathophysiology

Neurodegenerative diseases are characterized by the selective and progressive loss of neurons, leading to the development of neurological dysfunction which can include memory, behavioral, and movement deficits (Erkkinen et al., 2018). Neurodegenerative diseases include Alzheimer’s disease (AD), Parkinson’s disease (PD), frontotemporal dementia (FTD), amyotrophic lateral sclerosis (ALS), spinocerebellar ataxia’s, Huntington’s disease, multiple system atrophy, dementia with Lewy bodies, corticobasal degeneration, chronic traumatic encephalopathy, and prion diseases [e.g., Creutzfeldt-Jakob Disease (CJD), fatal familial insomnia, or kuru]. Collectively, these diseases exact a significant and growing toll on human well-being both socially and economically (Prince et al., 2013), making the development of effective therapies essential to societal health and well-being.

Although all of the above listed diseases have in common the progressive degeneration of neurons, they typically manifest clinically with different symptoms due to a characteristic set of neuronal subtypes specifically vulnerable in each condition (Erkkinen et al., 2018). For example, a patient suffering from canonical ALS will exhibit preferential degeneration of motor neurons in the spinal cord/brainstem and motor cortex, however, neurons in other central nervous system (CNS) regions will generally remain unaffected (Ravits and La Spada, 2009). Another example is that in PD dopaminergic neurons within the substantia nigra preferentially degenerate (Brichta and Greengard, 2014). Although the vulnerability of certain neurons to undergo death appears to be disease-specific, the key markers for this death are observed across many diseases (Chi et al., 2018). One such key marker is mitochondrial dysfunction, which can be a consequence of the accumulation of misfolded proteins like SOD1 (Magrané et al., 2012). Maintenance of mitochondrial protein homeostasis is especially important for neurons, owing to their large size and high energy requirements (reviewed in Baker et al., 2011).

A unifying feature of all the above mentioned neurodegenerative diseases is that the cell and neuroanatomical region specificity of degeneration is often accompanied by the aberrant misfolding, aggregation, and deposition of specific proteins (Chiti and Dobson, 2017). Protein misfolding is defined as the adoption of a non-native conformation by a protein; Protein aggregation is the aberrant accumulation of a protein into multimeric soluble or insoluble non-native structures; Protein deposition is the formation of large insoluble deposits of proteins (Chiti and Dobson, 2017). Examples of specific disease-associated aggregating proteins include amyloid-β (Glenner and Wong, 1984) and τ-protein (Grundke-Iqbal et al., 1986) in AD, α-synuclein in PD (Spillantini et al., 1997) and multiple system atrophy (Tu et al., 1998; Wakabayashi et al., 1998), poly-Q expansions in huntingtin protein in Huntington’s disease (DiFiglia et al., 1997), and either Cu/Zn superoxide dismutase (Rosen et al., 1993), TAR-DNA-binding protein of 43 kDa (TDP-43; Neumann et al., 2006), or fused in sarcoma (FUS; Kwiatkowski et al., 2009; Vance et al., 2009) in ALS.

It was previously thought that the aggregation of specific proteins is exclusively associated with specific disorders, however, there is evidence for some overlap between misfolding and aggregating proteins and various syndromes (Jellinger, 2012). Indeed, aberrant localization and aggregation of TDP-43 are being associated with an increasing number of neurodegenerative diseases (reviewed in Gao et al., 2018), and SOD1 positive inclusions have been reported in PD cases (Trist et al., 2017). Furthermore, the fact that patients suffering from FTD can have protein inclusion pathology that is positive for either TDP-43, τ-protein, or FUS (Ling et al., 2013), and that ~15% of ALS patients are estimated to develop cognitive deficits meeting FTD criteria (Ringholz et al., 2005), is striking evidence of both pathological and symptomatic overlap. This has led to the suggestion that both ALS and FTD perhaps exist as extreme syndromes of a single disease spectrum (Ling et al., 2013).

## Amyotrophic Lateral Sclerosis—Clinical and Pathological Manifestation

ALS is a neuromuscular disorder in which the upper (motor cortex) and lower (spinal) motor neurons, responsible for motor function, progressively degenerate, ultimately resulting in patient mortality *via* asphyxiation or inanition (Hardiman et al., 2017). The disease is considered to comprise two main categories on the basis of familial history of disease or lack thereof. Sporadic ALS (sALS) accounts for approximately 90% of cases (Taylor et al., 2016), and the remaining 10% are classed as familial (fALS). Collectively, ALS is estimated to have an annual incidence rate of 2.4 per 100,000 people in Europe, and is suggested to affect a susceptible population rather than being generally related with aging (Logroscino et al., 2010). Clinical diagnosis of ALS is often difficult due to significant variability in patient presentation and prognosis, as well as lack of a definitive biomarker for disease (Al-Chalabi et al., 2016; Grad et al., 2017). Generally, ALS patients will initially show symptoms of either lower motor neuron degeneration (weakness with muscle wasting, fasciculation, cramps) or upper motor neuron degeneration (weakness and spasticity), however, both regions of the CNS are ultimately affected (Tartaglia et al., 2007; Van den Berg-Vos et al., 2009). A characteristic feature of ALS is that often, regardless of the initial site of onset, symptoms spread to nearby contiguous anatomical regions in the CNS in a spatiotemporal manner (Ravits et al., 2007; Ravits and La Spada, 2009). There are cases in which the spread of symptoms has been reported to be discontiguous, however, they remain a minority (Walhout et al., 2018; Zhenfei et al., 2019). To better understand the relationship between symptoms and pathology, it will be important to examine discontiguous and contiguous spread in relation to the patterns of pathology in patients.

A key pathological hallmark of ALS is the deposition of proteins into ubiquitinated, and sometimes hyperphosphorylated, cytoplasmic inclusions in motor neurons and glia in the spinal cord/brainstem and motor cortex. Other hallmarks include the degeneration of motor neurons in the spinal cord and motor cortex, as well as atrophy of the corresponding denervated skeletal muscles. The pathological protein inclusions observed in patients are immunoreactive for either TDP-43 (Neumann et al., 2006), SOD1 (Rosen et al., 1993), or FUS (Kwiatkowski et al., 2009; Vance et al., 2009), of which the vast majority of cases show TDP-43 (97% of cases), rather than SOD1 (2%) or FUS (1%), pathology (Ling et al., 2013). Notably, aggregates of SOD1 or FUS are associated with mutations in cognate genes, whereas TDP-43 aggregates can be made from wild-type protein in sALS as well as associated with mutations in TDP-43 in fALS. TDP-43 pathology can also be observed as a downstream consequence of other ALS-implicated gene mutations (e.g., C9orf72).

## Genetics of ALS—Interplay of RNA Metabolism, Cytoskeletal Dynamics, and Proteostasis

Both sALS and fALS are clinically indistinguishable from each other (Hardiman et al., 2011), whereas the genetics of ALS are heterogeneous (Taylor et al., 2016), involving numerous genes that control various biochemical processes and cellular pathways (Taylor et al., 2016). As our understanding of the functions of these genes has increased, it has become apparent that they can be broadly classified into three major groups; genes associated with RNA metabolism, genes associated with cytoskeletal dynamics, and genes associated with protein homeostasis (proteostasis; Taylor et al., 2016). There is also a great deal of overlap between these important cellular processes, making it difficult to designate causal pathomechanism(s).

Disturbances in RNA metabolism in ALS have been keenly studied owing to the discovery that TDP-43 and FUS, which are RNA-binding proteins (RBPs), form neuronal inclusions and harbor mutations that are ALS causative (Neumann et al., 2006; Kwiatkowski et al., 2009; Vance et al., 2009). An increasing number of RNA metabolism-related genes have been identified to carry ALS-associated mutations, including heterogeneous nuclear ribonucleoprotein A1 (hnRNPA1) and heterogeneous nuclear ribonucleoprotein A2B1 (hnRNPA2B1; Kim et al., 2013), matrin-3 (Johnson et al., 2014), TIA-1 cytotoxic granule-associated RNA binding protein (Mackenzie et al., 2017), and TATA-box binding protein associated factor 15 (TAF-15; Couthouis et al., 2011; Ticozzi et al., 2011). Furthermore, the most common identified cause for fALS are hexanucleotide (GGGGCC) repeat expansions in the chromosome 9 open reading frame 72 (*C9orf72*) gene (DeJesus-Hernandez et al., 2011; Renton et al., 2011), that potentially lead to toxicity caused by the synthesis of large non-coding RNAs that form sense and anti-sense foci in cells that can sequester RBPs and prevent them from carrying out their biological functions (Donnelly et al., 2013; Gendron et al., 2013; Lagier-Tourenne et al., 2013; Mizielinska et al., 2013; Zu et al., 2013). Many of the above mentioned ALS-associated RBPs take part in the formation of ribonucleoprotein (RNP) granules and biomolecular condensates in cells (Banani et al., 2017), and there is growing evidence that mutations affect the dynamics of formation and dissolution of these structures (Murakami et al., 2015; Patel et al., 2015; Lee et al., 2016; Lin et al., 2016; Martinez et al., 2016; Boeynaems et al., 2017; Mackenzie et al., 2017). It is suggested that this dysregulation of RNP granules can result in significant alterations to alternative splicing, mRNA transcription, and RNA transport, which can result in widespread toxicity in cells (Butti and Patten, 2019). An overlap with proteostasis mechanisms is also possible with ALS-associated mutations in RBPs as they can act to promote a liquid-to-solid transition of biomolecular condensates, leading to the formation of pathological aggregates in cells (Murakami et al., 2015; Patel et al., 2015; Mackenzie et al., 2017; Sun and Chakrabartty, 2017; Gasset-Rosa et al., 2019). Cytoskeletal dynamics can also be affected by ALS-associated RBP mutants, for example, ALS-associated mutations in TDP-43 can alter the transport dynamics and viscosity of transport RNPs (Alami et al., 2014; Gopal et al., 2017), potentially resulting in impaired delivery of mRNA to the axons of motor neurons.

Motor neurons are significantly larger than other cell types, owing to the requirement to extend axons anatomically long distances throughout the motor cortex, spinal cord and periphery. Therefore, disruption of the mechanisms that traffic protein and nucleotides to distal cellular compartments would be expected to impair proper cellular functioning. Indeed, ALS-associated mutations have been identified in cytoskeletal components and regulatory elements, such as heavy neurofilament (Al-Chalabi et al., 1999), peripherin (Gros-Louis et al., 2004), NIMA-related kinase 1 (NEK1; Kenna et al., 2016), profilin-1, and kinesin family member 5A (KIF5A; Brenner et al., 2018), amongst others (Puls et al., 2003; Wu et al., 2012; Smith et al., 2014). The mechanisms by which mutations in cytoskeletal components can cause ALS are not fully understood but are thought to be a combination of loss-of-function and gain-of-toxic-function that can overlap with both RNA metabolism and proteostasis (Hensel and Claus, 2017). In the case of profilin-1, in its normal role it binds to actin and it affects the overall structure of the cytoskeleton. In terms of RNA metabolism, it was found that ALS-associated profilin-1 mutants differentially localized to stress granules and that stress granule dynamics were altered (Figley et al., 2014). In the case of proteostasis, expression of ALS-associated profilin-1 mutants has been shown to lead to the formation and seeding of TDP-43 positive proteinaceous aggregates in cultured cells (Wu et al., 2012; Smith et al., 2015; Tanaka and Hasegawa, 2016).

That proteinaceous aggregates are the primary characteristic marker for ALS suggests that, at the very least, proteostasis is a key mechanism affected in ALS. Indeed, there are several identified ALS-associated mutations in genes associated with autophagy (Maruyama et al., 2010; Fecto et al., 2011), and the ubiquitin proteasome system (UPS; Johnson et al., 2010; Deng et al., 2011; Williams et al., 2016). Furthermore, autophagy and the UPS have been suggested to be altered in sALS on the basis of studies of post-mortem tissue (Sasaki, 2011; Kabashi et al., 2012). Interestingly, TDP-43, SOD1, and FUS, which are the primary components of inclusions in ALS patient tissue (Ling et al., 2013), are shown to be supersaturated (expressed at levels higher than their predicted solubility) in spinal motor neurons (Ciryam et al., 2017). Following this notion, it has been suggested that the average supersaturation of the entire proteome of spinal motor neurons is greater than that of the ALS-resistant ocular motor neurons (Yerbury et al., 2019), providing important physico-chemical insight into the selective vulnerability of spinal motor neurons to proteotoxicity. Again, overlap is observed between proteostasis and both RNA metabolism and cytoskeletal dynamics. For example, the UPS protein ubiquilin-2 has been observed to control the dynamics of both stress granule formation and RNA/FUS complexes (Alexander et al., 2018), and mutant SOD1 can interact with the stress granule forming protein Ras GTPase-activating protein-binding protein 1 (G3BP1) to impair proper stress granule formation (Gal et al., 2016).

Collectively, RNA metabolism, cytoskeletal dynamics, and proteostasis should not be viewed as separate pathologically altered pathways, but as an interconnected network. However, regardless of whether RNA metabolism, cytoskeletal dynamics, or proteostasis are perturbed in ALS, the most prominent pathology is the formation of proteinaceous aggregates in neurons and glial support cells composed of TDP-43, or SOD1, or FUS in a mutually exclusive manner (Farrawell et al., 2015). This is supported by the finding that TDP-43 and FUS, despite both being RNA binding proteins participating in stress granule formation, generally do not appear to co-localize in pathological ALS inclusions (Ling et al., 2013). Furthermore, the neuroanatomical spread of pathology and symptoms indicates that there are propagating agents in ALS (Table 1). Owing to the spatiotemporal spread of pathology in ALS, as well as PD and AD, it has been suggest that a key mechanism in neurodegenerative disease pathobiology is the “prion-like” spread of protein misfolding and aggregation, pathways of which would be different for the three major inclusion forming proteins (Farrawell et al., 2015) to account for the exclusivity noted above.

**Table 1:** Evidence supporting the prion-like characteristics of ALS-associated proteins.

Protein/Gene	*in vitro* fibril formation	*in vitro* fibril seeding	Cell seeding with *in vitro* protein	Cell-to-cell propagation	Human to cell propagation	*in vitro* to animal propagation	Animal to animal propagation	Human to animal propagation
SOD1	✓	✓	✓	✓	✓	✓	✓	✓
	Chattopadhyay et al. (2008)	Chia et al. (2010)	Münch et al. (2011)	Grad et al. (2014)	Pokrishevsky et al. (2017)	Ayers et al. (2016a)	Ayers et al. (2014)	Ekhtiari Bidhendi et al. (2018)
TDP-43	✓	✓	✓	✓	✓	–	–	✓
	Johnson et al. (2009)	Furukawa et al. (2011)	Furukawa et al. (2011)	Nonaka et al. (2013)	Nonaka et al. (2013)			Porta et al. (2018)
FUS	✓	✓	–	–	–	–	–	–
	Nomura et al. (2014)	Nomura et al. (2014)
C9orf72 (DPRs)	–	–	–	✓	–	–	–	–
				Westergard et al. (2016)				

*Note: ✓ = confirmed; – = no published evidence*.

## Prion Disease and Prion Theory

Classical understanding of progressive and infectious diseases mandated that a nucleic acid component be present to facilitate replication of the infectious particles. This idea has since been challenged owing to the discovery that a class of neurological disorders, known as the prion diseases, possess an infectious life cycle and spread in the CNS in the absence of any microbial nucleic acid component. The landmark finding was that the infectious particle responsible for scrapie, a neurodegenerative disease that affects sheep and goats, was proteinaceous in origin, resulting from the misfolding of prion protein (PrP^C^) into a pathological conformation (PrP^Sc^; Prusiner, 1982). Furthermore, PrP^Sc^ was found to be able to catalyze the misfolding of PrP^C^, thus generating more conformational copies of PrP^Sc^ that can then spread throughout cells, tissues, and even between organisms. This notion of the propagation of protein misfolding has been subsequently applied to neurodegenerative diseases, providing a possible explanation for the protein aggregation pathology and progressive spatiotemporal degeneration observed in these diseases.

Prion diseases include CJD, Bovine Spongiform Encephalopathy, Fatal Familial Insomnia, Kuru, and Gerstmann–Sträussler–Scheinker disease, amongst others (Geschwind, 2015). The clinical manifestations and pathological profiles of prion diseases are highly similar to those of other neurodegenerative diseases (Prusiner, 2001). In both cases they are invariably fatal; The clinical symptoms are mainly associated with neurological functioning and can include dementia, ataxia, involuntary movements, weakness, and spasticity, although, specific prion diseases will manifest distinct symptoms from this list (Glatzel et al., 2005). The neuropathological similarities of prion diseases with other neurodegenerative diseases include substantial neuronal loss, accumulation of proteins into aggregates, gliosis, and general cerebral atrophy (Prusiner, 2001).

Biophysical examination of PrP^C^ has suggested that its aggregation is preceded by a conformational change from a structure composed mostly of α-helical elements to a β-sheet rich conformation, where the β-sheet rich conformation is thought to be the disease-associated PrP^Sc^ form (Pan et al., 1993). The β-sheet rich form is capable of interacting with and converting the normal α-helix rich form to the β-sheet rich form in a template-directed manner. Furthermore, the β-sheet rich form of PrP is capable of polymerizing into amyloid-like fibrils (McKinley et al., 1991). This is a similar kinetic process that occurs to amyloid-β peptides to generate amyloid fibrils (Arosio et al., 2015). The process is understood as an initial monomeric misfolding event, followed by the formation of small disordered oligomers, where oligomers nucleate the formation of the larger ordered amyloid fibrils. Interestingly, *in vitro* studies have shown that many neurodegenerative disease-associated proteins are capable of forming amyloid-like fibrils (Eisenberg and Jucker, 2012; Knowles et al., 2014). In ALS this includes TDP-43 (Chen et al., 2010; Guo et al., 2011; Saini and Chauhan, 2011, 2014; Jiang et al., 2013; Wang et al., 2013; Zhang et al., 2013; Mompeán et al., 2014; Sun et al., 2014; Cao et al., 2019), SOD1 (Chattopadhyay et al., 2008, 2015; Münch and Bertolotti, 2010; Lang et al., 2012; Chan et al., 2013; Ivanova et al., 2014; Abdolvahabi et al., 2016; McAlary et al., 2016), FUS (Kato et al., 2012; Murakami et al., 2015; Patel et al., 2015; Murray et al., 2017; Luo et al., 2018), amongst others (Molliex et al., 2015; Mackenzie et al., 2017).

Within the schema of prion theory, there exists a notion that different structural conformations of the prion agent may confer specific modes of spread, different abilities to recruit normal protein, and different levels and mechanisms of cellular toxicity to recipient species (Morales, 2017). Together, these characteristics are used to define different “strains” of prion. An integral part of the prion strain idea is that the ability of a prion strain to transmit disease is dependent on the conformation of the prion particle, the amino acid sequence of the template-competent substrate, post-translational modification of the substrate, and the cell type (substrate expression level and cellular capacity for degradation of misfolded/aggregated protein; Telling et al., 1996; Safar et al., 1998; Legname et al., 2006). Conformation of the prion assembly is especially important, as it has been established that strains that are more likely to fragment into a greater number of seeds are the most capable of spreading (Tanaka et al., 2006). In relation to ALS, it is interesting to note that the main aggregating proteins (SOD1, TDP-43, FUS) are supersaturated in spinal motor neurons (Ciryam et al., 2017), suggesting that motor neurons may provide an efficient environment for ALS-associated prion-like seeds to form and propagate. Indeed, the combination of the spatiotemporal spread of symptoms in ALS with both the evidence of protein aggregation and ability of the aggregating proteins to form amyloid-like structures has led to the suggestion that a prion-like mechanism is active in ALS (Ayers and Cashman, 2018). Below we review the evidence for prion-like characteristics of SOD1, TDP-43, FUS, and *C9orf72* associated dipeptide repeats, as they relate to ALS.

## Superoxide Dismutase-1

### SOD1 Structure and Function

**Figure 1:**
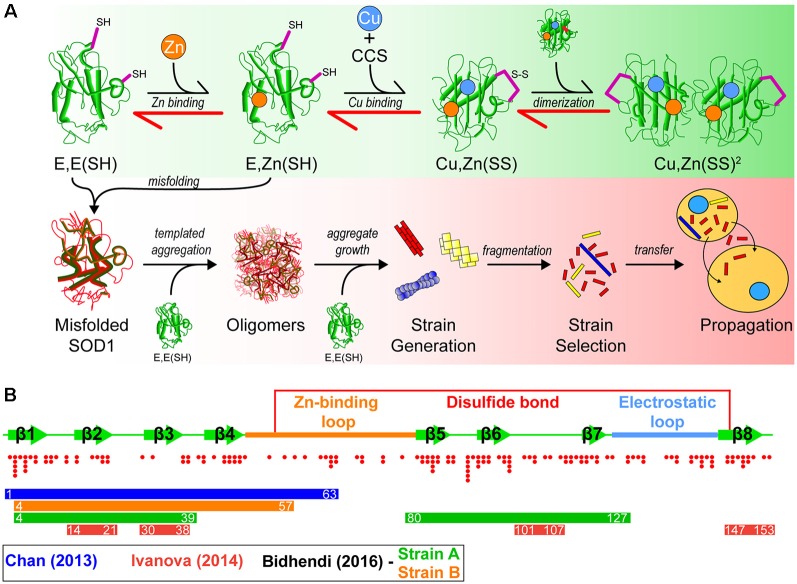
A model of superoxide dismutase-1 (SOD1) aggregation and prion-like propagation. **(A)** The native folding (green background) and off-folding pathways (red background) of SOD1. When undergoing native folding, SOD1 is initially in a metal-free intermediate state [E, E(SH)] which is primed for Zn binding. Following Zn binding, the copper chaperone for SOD1 (CCS) heterodimerizes with a SOD1 monomer to implant Cu and facilitate the oxidation of the disulfide bond between Cys57 and Cys146 to form a mature SOD1 monomer [Cu, Zn(SS)]. Subsequent dimerization of two mature monomers gives the fully folded SOD1 enzyme. In the off-folding pathway (red background) amyotrophic lateral sclerosis (ALS)-associated mutations have the ability to push the folding back towards the nascent intermediates (red arrows), which are more aggregation prone than mature states. Misfolded SOD1 is capable of recruiting nascent SOD1 states to form oligomers. Oligomers can grow in size to form larger aggregates that may have strain-like properties. Strains that are prone to fragment are the most likely to propagate to adjacent cells and seed naïve SOD1. **(B)** Graphic representation of the SOD1 primary structure with important features. SOD1 is composed of 8 beta-strands and contains two major loop regions. The Zn-binding loop is responsible for coordination of Zn and, to a lesser extent, Cu. The electrostatic loop is responsible for guidance of superoxide substrate to the active site of the enzyme. A disulfide bond is formed between Cys57 and Cys146 in the mature protein. Red dots below the graphic represent the location and number of ALS-associated mutations that have been identified to date within SOD1. Coloured bars represent potential cores or contributing regions of the protein to the formation of aggregate strains from corresponding colour coded publications.

Superoxide dismutase-1 (SOD1) was the first gene linked to familial ALS (Rosen et al., 1993), and since this initial discovery, there have been over 160 fALS-associated mutations identified in the *Sod1* gene (Abel et al., 2013). In its natively folded state, SOD1 is a 32 kDa homodimer, where each 153 amino acid subunit coordinates a zinc ion, copper ion, and contains an intramolecular disulfide bond. The maturation pathway (Figure 1A) of SOD1, from nascent polypeptide to native dimeric conformation, is complex for such a small protein and has been extensively studied (Banci et al., 2012, 2013; Wright et al., 2016; Luchinat et al., 2017; Sala et al., 2019). Following synthesis, SOD1 assumes a partially folded state with no metal cofactors and reduced cysteines [E, E(SH)]. This nascent state is further stabilized by the binding of a zinc (Zn) ligand to form E, Zn(SH). The copper chaperone for SOD1 (CCS) then associates with the Zn-bound intermediate monomer, forming a heterodimer that facilitates Cu delivery and intramolecular disulfide formation to form a mature SOD1 monomer [Cu, Zn(SS)]. Interestingly, this interaction can be prohibited or stalled by SOD1-fALS mutations (Wright et al., 2016). Disulfide formation *via* CCS can also occur through a Cu-independent pathway (Banci et al., 2013) and can be promoted using small molecules (Capper et al., 2018). Recent work has also suggested that CCS performs a chaperone function to stabilize E, E-SOD1^2SH^ SOD1 and promote Zn-binding mediated by its own SOD-like domain (Luchinat et al., 2017). The final step is dimerization of mature monomers to form the highly stable enzyme. Once natively folded, SOD1 acts to catalyze the dismutation of superoxide anions to hydrogen peroxide and molecular oxygen.

### Loss of Function or Gain of Toxic Function?

Owing to the role of SOD1 as an antioxidant enzyme, it was initially thought that the fALS-associated mutations in SOD1 were inducing a loss of enzymatic function, leading to toxicity mediated by increased concentrations of oxygen radicals in cells. However, continued research revealed that this was not the case as overexpression of human mutant SOD1-G93A in a transgenic mouse line showed increased dismutase activity combined with rapid mortality in comparison to wild-type mice (Gurney et al., 1994). Another study knocked out SOD1 from mice, showing slightly increased neuronal cell death following axonal injury, but normal motor neuron development (Reaume et al., 1996), indicating that SOD1 is not essential to cell survival or development, and its antioxidant activity may be compensated for by other proteins (including SOD2 and SOD3). Furthermore, *in vitro* studies of several SOD1-fALS mutants found no correlation between relative enzymatic activity and clinical pathology (Borchelt et al., 1995; Ratovitski et al., 1999), and that many SOD1-fALS mutants possess significant enzymatic activity (Borchelt et al., 1994). Ruling out a loss-of-function mechanism for SOD1-fALS led to research into a gain-of-toxic-function mechanism, focused on the misfolding and aggregation of SOD1 (Figure 1A).

### SOD1 Pathological Features in Patients

Immunohistochemical studies of post-mortem tissue from both fALS and sALS patients with SOD1 misfolding-specific antibodies have revealed a potential role for the misfolding of WT SOD1 in all ALS (Bosco et al., 2010; Forsberg et al., 2010, 2011, 2019; Pokrishevsky et al., 2012; Paré et al., 2018), however, this is a contentious idea (Liu et al., 2009; Kerman et al., 2010; Da Cruz et al., 2017). Regardless, discrete SOD1 inclusions are a key component of fALS with SOD1 mutations. Currently, there are discrepancies as to the exact structural nature of the SOD1 inclusions found in patient tissue. Electron microscopy imaging of protein inclusions harvested from ALS patients suggests that they are distinct from classic amyloid, having an amorphous or granular structure (Kato et al., 2000) that does not stain with Congo red, and stains relatively weakly with thioflavin-S (Kerman et al., 2010). Whether amyloid-like elements exist in inclusions composed of SOD1 in SOD1-fALS patients remains to be fully elucidated, although it is important to note that SOD1 has the ability to form amyloid-like fibrils *in vitro* (Chattopadhyay et al., 2008, 2015; Münch and Bertolotti, 2010; Lang et al., 2012; Chan et al., 2013; Ivanova et al., 2014; Abdolvahabi et al., 2016; McAlary et al., 2016). Furthermore, aggregated SOD1 in human mutant SOD1-expressing transgenic mice is observed to have an ordered microscopic and molecular structure (Ayers et al., 2014, 2016b; Bergh et al., 2015; Lang et al., 2015; Bidhendi et al., 2016, 2018).

### Amyloid Aggregation of SOD1

The first evidence that SOD1 may act in a prion-like manner came from protein only *in vitro* assays utilizing recombinant wild-type (WT) SOD1 and fALS-mutants. Initially, SOD1 aggregation assays utilized high temperatures, extreme pH, and organic solvents to induce aggregate assembly (DiDonato et al., 2003; Stathopulos et al., 2003; Banci et al., 2008), conditions with little relevance to the intracellular environment. Modern recombinant SOD1 *in vitro* aggregation protocols rely upon destabilizing SOD1 *via* reduction of the disulfide and chelation of the metals under physiological salt and pH (Chattopadhyay et al., 2008; Lang et al., 2012; Chan et al., 2013; Ivanova et al., 2014; Abdolvahabi et al., 2016; McAlary et al., 2016), which are events more likely to occur in the reducing and metal-poor intracellular environment. Utilizing denaturing conditions, Chia et al. (2010) demonstrated that preformed recombinant SOD1 fibrils or spinal cord homogenates from multiple SOD1 transgenic mice were capable of seeding the aggregation of recombinant WT or mutant Cu, Zn-SOD1^SS^ *in vitro*. This requirement to destabilize SOD1 state to promote fibril formation and seeding suggests that immature SOD1 may be the major molecular species prone to aggregation and a primary template for SOD1 seeded aggregation. Indeed, careful investigations into the *in vivo* aggregation of SOD1 mutants in transgenic mice have been shown to have a striking similarity to these cell-free recombinant systems, suggesting E, E(SH) SOD1 is the primary aggregation substrate, and that aggregate growth is fragmentation dependent both *in vivo* and *in vitro* (Lang et al., 2015).

Interestingly, SOD1 has multiple sequence segments that have characteristics favorable to form amyloid fibrils (Ivanova et al., 2014). These include amino acid sequence segments _14_VQGIINFE_21_, _30_KVWGSIKGL_38_, _101_DSVISLS_107_, and _147_GVIGIAQ_153_, as determined by using the 3D profile method (see Münch and Bertolotti, 2010). The 3D profile method uses a sliding window of every six amino acid peptides from a queried protein sequence to generate structures of these peptides, where these peptide structures are computationally mapped onto ensembles created from the amyloidogenic NNQQNY peptide sequence from Sup35 yeast prion-protein. Peptides with strong binding energies are considered to be potentially amyloidogenic (Thompson et al., 2006). Assays on these synthesized peptides have shown that all have the ability to form fibrils *in vitro*, and seed full-length SOD1 aggregation (Ivanova et al., 2014). Of particular note is segment _30_KVWGSIKGL_38_ which has been recently shown to be able to form oligomers that are neurotoxic (Sangwan et al., 2017, 2018), and also contains the only tryptophan residue in SOD1 (W32), which is implicated in its aggregation and prion-like propagation (Taylor et al., 2007; Grad et al., 2011, 2014; Pokrishevsky et al., 2018). That SOD1 can form fibrils which may be nucleated by several different regions of the protein or controlled by specific residues, has implications for the existence of SOD1 prion-like strains (Figure 1B).

### Intra- and Inter-cellular Prion-Like Spread of SOD1 Aggregation

The ability of cellular SOD1 to misfold and/or aggregate in a prion-like manner in living cells was first demonstrated using the addition of recombinant SOD1 aggregates to cultured cells overexpressing ALS mutant SOD1 variants (Münch et al., 2011). It was suggested that the exogenous SOD1 aggregates were entering the cells *via* macropinocytosis and escaping the macropinosomes to seed aggregation of cytosolic SOD1 (Münch et al., 2011), a finding that was substantiated in following publications (Holmes et al., 2013; Grad et al., 2014; Zeineddine et al., 2015). Shortly following the first study reporting intracellular seeding of SOD1, another study demonstrated the ability of endogenous wild-type SOD1 to become misfolded when ALS-associated mutant SOD1 was overexpressed in cultured cells (Grad et al., 2011). It was also determined that the prion-like propagation of SOD1 misfolding required the unique tryptophan at position 32 in both seed and substrate molecules (Grad et al., 2011). However, although a potential mechanism of cellular uptake and seeding was suggested, it was not known if or how aggregated or misfolded SOD1 may exit cells and spread. Grad et al. (2014) demonstrated the intercellular propagation of SOD1 misfolding and aggregation, and established two main mechanisms by which spread was occurring: release of aggregates from dying cells, and/or packaging of misfolded protein seed into or on extracellular vesicles called exosomes. More recently, it has been shown that extracellular vesicles purified from the neural tissues of SOD1-G93A mice carry misfolded SOD1 (Silverman et al., 2019), providing further evidence that this mechanism may play an important role in prion-like propagation of protein aggregation in ALS. Additionally, homogenates generated from spinal cord samples of SOD1-fALS patients were recently found to seed the aggregation of ALS mutant SOD1-GFP protein expressed in human embryonic kidney cells (Ayers et al., 2014; Pokrishevsky et al., 2017).

### *In vivo* Seeding and Spread of SOD1 Aggregation

Evidence showing the prion-like propagation of SOD1 *in vivo* shortly followed the cultured cell experiments, providing information on the existence of different transmissible strains of SOD1. Initially, transgenic mice expressing low levels of the SOD1-G85R ALS mutant C-terminally tagged with yellow fluorescent protein (SOD1-G85R:YFP) were used as a model owing to them not developing SOD1-associated ALS-like pathology or symptoms when the transgene was heterozygous (Wang et al., 2009). Spinal cord homogenates harvested from mice overexpressing SOD1 mutants were injected into the sciatic nerve, cervical spinal cord, or cerebral ventricles of SOD1-G85R:YFP mice in an attempt to induce ALS-like pathology and symptoms (Ayers et al., 2014). Potent seeding was observed following injection into the mice, resulting in the formation of SOD1-G85R:YFP aggregates in neurons in spinal cord and motor cortex. The formation of SOD1-G85R:YFP aggregates was accompanied by the typical ALS phenotypes of motor dysfunction and paralysis. Later, this same model was used to examine the anatomical pathways by which SOD1 aggregates may traverse the CNS, finding that aggregation spread through synaptically connected cells in the spinal cord (Ayers et al., 2016b), providing useful information on possible mechanisms of prion-like transmission in ALS.

Considering that slight differences in the conformation of PrP^Sc^ can result in different pathological activities in prion diseases (Prusiner, 2013), it has been suggested that this could also occur in regards to SOD1 aggregation in fALS. Evidence for the existence of SOD1 strains *in vivo* was first shown by binary epitope mapping the insoluble material from spinal cords of transgenic mice overexpressing WT, G85R, D90A, or G93A SOD1 variants (Bergh et al., 2015), in which two strains (denoted strain A or strain B) were identified on the basis of differential epitope exposure. Mice expressing WT, G85R, and G93A were found to contain strain A aggregates, whereas D90A expressing mice contained either strain A or B aggregates (Bergh et al., 2015). Additionally, strain B aggregates correlated with a more rapid disease progression in mice and were more structurally unstable than strain A aggregates, suggesting aggregate fragmentation as an important mechanism in prion-like spreading (Bergh et al., 2015; Lang et al., 2015). These strains were found to be transmissible to SOD1-G85R transgenic mice through injection of fractions enriched with insoluble SOD1 from strain A or B into the spinal lumbar spinal cord, resulting in strain propagation as measured by binary epitope mapping and ALS-like symptoms (Bidhendi et al., 2016). Similar results were also obtained using the aforementioned SOD1-G85R:YFP mice, where injections of recombinant WT SOD1 protein resulted in aggregates with a thread-like morphology in recipient mice, in comparison to more amorphous features from injections using homogenates from other SOD1-fALS mice (Ayers et al., 2016a). More recently, transmission of strain A was found to occur when enriched insoluble material from the spinal cord of a SOD1-G127X patient was injected into transgenic mice harboring the same mutation (Ekhtiari Bidhendi et al., 2018). Collectively, the combined cell-free, *in vitro*, and *in vivo* studies strongly suggest that SOD1 acts in a prion-like manner in SOD1-fALS.

## TDP-43 Prion Evidence

### TDP-43 Structure and Function

**Figure 2:**
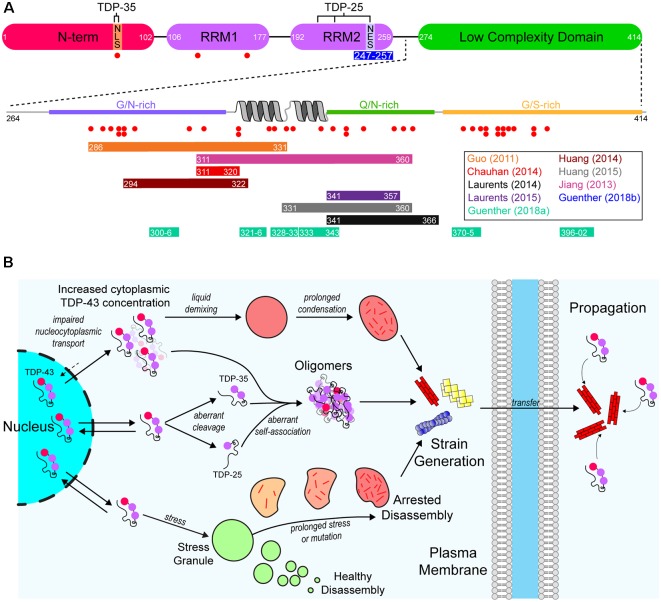
A model of the aggregation and prion-like propagation of TAR DNA-binding protein of 43 kDa (TDP-43) in ALS. **(A)** TDP-43 is composed of four domains which include the ubiquitin-like N-terminal domain (NTD), tandem RNA-recognition motifs (RRM), and a C-terminal low complexity domain (LCD). The NTD contains the nuclear localization signal (NLS) of TDP-43, whereas the nuclear export signal (NES) is in RRM2. The pathological 35 kDa (TDP-35) and 25 kDa (TDP-25) are formed *via* aberrant caspase-mediated cleavage at the NLS and in RRM2 respectively. The blue bar below RRM2 represents an amyloidogenic segment. The LCD is a prion-like domain due to it being enriched for G/N/Q/S residues. It also contains a helix-turn-helix motif which is important for association with other TDP-43 LCDs. Red dots below both the main TDP-43 graphic and the LCD expansion represent ALS-associated mutations. Colored bars represent experimentally identified regions of the protein that are capable of forming amyloid-like structures. Bar colors correspond to the publications listed on the right. **(B)** TDP-43 is a primarily nuclear-localized protein, however, it shuttles between the nucleus and cytoplasm. Impairment of nucleocytoplasmic transport (top) can lead to the cytoplasmic concentration of TDP-43 increasing, leading to liquid demixing to form biomolecular condensates distinct from stress granules. Prolonged residency of TDP-43 in these condensates can lead to the formation of aggregates. Aberrant cleavage (middle) leads to the formation of the highly aggregation-prone TDP-35 and TDP-25 pathological fragments. These fragments, and even full-length TDP-43 can aberrantly self-associate to form oligomers that may grow to become larger aggregates. Also, TDP-43 is known to inhabit stress granules (bottom). Given prolonged stress or genetic mutations, stress granules can persist for longer than necessary and can fail to disassemble, which leads to the formation of aggregates. Each of these possible pathways may also result in strains of TDP-43 aggregates that can propagate from cell-to-cell and seed further aggregation.

As mentioned previously, aggregated cytoplasmic TDP-43 is observed in the majority of ALS cases (Ling et al., 2013). TDP-43 is a DNA/RNA-binding protein that has various roles in RNA metabolism, including trafficking, splicing, and degradation (Buratti et al., 2001, 2005; Hefferon et al., 2004; Mercado et al., 2005). The protein contains both a nuclear export signal (NES) and a nuclear localization signal (NLS), however, it is predominantly localized to the nucleus. Structurally, the protein is composed of 4 domains; an N-terminal domain (NTD) with a ubiquitin-like fold (Mompeán et al., 2016), tandem RNA-recognition motifs (RRM), and a prion-like (on the basis of sequence properties similar to yeast prion proteins) C-terminal low-complexity domain (LCD) rich in Gln/Asn residues (Figure 2A). The architecture of TDP-43 plays a significant role in its ability to interact with other TDP-43 molecules and other proteins. For example, the NTD can mediate the formation of physiological TDP-43 multimers (Afroz et al., 2017; Mompeán et al., 2017; Tsoi et al., 2017; Wang et al., 2018), and the LCD mediates TDP-43’s ability to form or take part in biomolecular condensates (Conicella et al., 2016; Li et al., 2018), such as stress granules (Bentmann et al., 2012).

### TDP-43 Pathological Features in Patients

Pathologically, TDP-43 is observed to be mislocalized from the nucleus to the cytoplasm in degenerating neurons, where it forms large ubiquitinated and hyperphosphorylated cytoplasmic inclusions (Neumann et al., 2006). Although there is some debate, strong evidence indicates that at least some of the TDP-43 inclusions observed in patient tissue contain amyloid structures (Mori et al., 2008; Bigio et al., 2013; Nonaka et al., 2013; Porta et al., 2018; Laferrière et al., 2019). Indeed, immunohistochemical examination of sALS patient tissue suggests that maturation of some TDP-43 inclusions proceeds first through small linear “wisps” in the cytoplasm that then go on to form thicker thread-like aggregates, which then grow to form skein-like inclusions (Mori et al., 2008). Additionally, more exhaustive processing of patient tissue to reduce background fluorescence shows that TDP-43 inclusions in ALS are strongly thioflavin-S fluorescent (Bigio et al., 2013). Furthermore, inclusions in sALS patient tissue that are observed to be non-linear at the micron scale are found to have a linear filamentous structure at the scale of tens of nanometers (Lowe et al., 1988; Lowe, 1994), suggesting potential amyloid-like aggregation is associated with TDP-43-related ALS. Biochemical characterization of the insoluble fractions of ALS patient CNS tissue showed that TDP-43 is aberrantly cleaved into 35 kDa and 25 kDa C-terminal fragments (Neumann et al., 2006) which are aggregation-prone and toxic in cell overexpression models (Igaz et al., 2009; Zhang et al., 2009; Yang et al., 2010; Nonaka et al., 2013). Although the TDP-43 C-terminal fragments are associated with ALS and FTD, what causes their fragmentation and what their pathological role might be is still debated (reviewed in Berning and Walker, 2019). It is interesting to note that genetic screening has identified more than 50 mutations in TDP-43 that are ALS or FTD-associated, with most of these mutations being localized to the LCD (Abel et al., 2013), a domain which comprises the bulk mass of the pathological C-terminal fragments.

### Amyloid Aggregation of TDP-43

Regarding the aggregation propensity of TDP-43, the LCD is of particular interest since it is predicted to be a prion-like domain (PrLD) on the basis of similarity to the yeast prion protein Sup35 sequence physicochemical properties, which is enriched for asparagine, glutamine, tyrosine and glycine residues (King et al., 2012). Some PrLDs are capable of forming amyloid owing to their amino acid sequence being enriched for polar and uncharged amino acids such as glycine, glutamine, tyrosine, and asparagine (Alberti et al., 2009; Toombs et al., 2010; King et al., 2012). Indeed, application of the 3D-profile method (Thompson et al., 2006) to the TDP-43 LCD identified several regions that could potentially form amyloid fibrils, with subsequent crystallography experiments showing that 6 of these segments (_300_GNNQGSN_306_, _321_AMMAAA_326_, _328_AALQSS_333_, _333_SWGMMGMLASQ_343_, _370_GNNSYS_375_, and _396_GFNGGFG_402_) could form steric zippers (Guenther et al., 2018a), a structure that is characteristic of the spines of amyloid fibrils (Sawaya et al., 2007; Eisenberg and Jucker, 2012). Other studies, using longer peptide segments, have also identified amyloid-forming regions within the TDP-43 LCD (Chen et al., 2010; Guo et al., 2011; Jiang et al., 2013, 2016; Mompeán et al., 2014; Cao et al., 2019; Figure 2A). A recent study has used cryo-electron microscopy to determine the structure of several TDP-43 LCD polymorphic fibrillar assemblies occurring from two segments of the LCD corresponding to amino acid residues 311–360 and 286–331 (Cao et al., 2019). This has implications for the prion-like nature of TDP-43 as different fibrillar polymorphs may have different prion-like characteristics.

Other than the LCD, a segment of RRM2 (_247_DLIIKGISVHI_257_) has been shown to form several steric zipper polymorphs (Guenther et al., 2018b), suggesting this domain may also contribute to pathological aggregation at least when RRM2 is partially folded (Tavella et al., 2018). Additionally, the size of the TDP-43 fragments (35 kDa or 25 kDa) influences the aggregation and degradation pathways of TDP-43 (Kasu et al., 2018). On the other hand, the majority of evidence for TDP-43 to form amyloid comes from purified protein studies utilizing C-terminal fragments that are either pathological fragments (35 or 25 kDa) or just the LCD (amino acids 274–414), as well as just the RRMs or small segments thereof (Guenther et al., 2018b; Agrawal et al., 2019; Cao et al., 2019). Less work has been carried out examining the ability of full-length TDP-43 to form amyloid in these assays (Furukawa et al., 2011; Vogler et al., 2018), which may be due to the difficulty in purifying and handling full-length recombinant TDP-43. It appears as though the full-length protein is capable of forming amyloid at low micromolar concentrations (Furukawa et al., 2011), and also capable of being seeded by cell culture-derived TDP-43 positive myo-granules (Vogler et al., 2018). Collectively, the above evidence suggests that, at the least, the pathologic fragments of TDP-43 are capable of forming amyloid and that the LCD makes a significant contribution to the formation of TDP-43 amyloid-like fibrils (Figure 2A).

### Contribution of Phase Transitions to TDP-43 Amyloid Formation

Considering the importance of the TDP-43 LCD to the formation of both amyloid (Chen et al., 2010; Guo et al., 2011; Jiang et al., 2013, 2016; Mompeán et al., 2014) and biomolecular condensates (Conicella et al., 2016; Li et al., 2018), it is interesting to consider the relationship between these phenomena. Previous studies investigating ALS-associated mutations in both FUS (Murakami et al., 2015; Patel et al., 2015) and hnRNPA1 (Molliex et al., 2015) have highlighted their capacity to mature/age from a liquid-like state to more solid gels composed of fibrillar structures under conditions that promote phase transition. In the case of TDP-43, recent work has suggested that phase transitions of purified TDP-43 LCD promote the formation of amyloid-like fibrils (Babinchak et al., 2019), however, it is important to note that in some cases fibrils generated from the phase transition of PrLDs can be sensitive to sodium-dodecyl-sulfate (SDS; Lin et al., 2015), whereas typical amyloid is resistant to SDS denaturation (Kryndushkin et al., 2003; Halfmann and Lindquist, 2008). Indeed, recent work has highlighted the contribution of small sequence segments containing aromatic residues within PrLDs, called “low-complexity aromatic-rich kinked segments” (LARKS), to reversible phase transition and fiber formation (Hughes et al., 2018). Importantly, LARKS can form fibers that are calculated to have weaker binding energies than the steric zippers that form amyloid spines, evidenced by the LARKS-formed fibrils being susceptible to dissolution by gentle heat (Hughes et al., 2018). It has been suggested that post-translational modification or mutation of the TDP-43 LCD can result in the strengthening of LARKS interactions to become irreversible (Guenther et al., 2018a), although these experiments were performed using small peptide segments of the TDP-43 LCD. It remains to be established if full-length TDP-43 that has undergone maturation in its liquid-liquid phase-separated state forms typical steric zipper amyloid structures, although it is highly likely.

Cell culture studies of the ability of TDP-43 to form biomolecular condensates, and examination of the consequences of condensate formation, have been difficult for several reasons. First, the induction of stress granules requires stressors such as oxidative stress, heat stress, or osmotic stress (Aulas et al., 2017). These stressors not only affect cell health, making the long term monitoring of condensate maturation into aggregates difficult but also induce condensates with different protein constituents dependent on the stressor or cell type used (Aulas et al., 2017). Methods to overcome this have recently been introduced with the usage of light-sensitive proteins to promote protein-protein interactions of proteins capable of undergoing phase transitions (Shin et al., 2017, 2018; Dine et al., 2018; Bracha et al., 2019; Zhang et al., 2019). Replacement of the dimerization domain on G3BP1 with the light-sensitive Cryptochrome-2 (CRY2) protein allowed for the light-inducible generation of biomolecular condensates with similar biochemical and physical properties to stress granules without the addition of stressors (Zhang et al., 2019). The authors found that repetitive or sustained induction of the light-inducible condensates resulted in their evolution to inclusions with the pathological hallmarks of ALS, such as the deposition of SQSTM1 and the hyperphosphorylation of TDP-43 (Zhang et al., 2019). This work suggests that the initial site of TDP-43 aggregate nucleation may be in stress granules or perhaps another biomolecular condensate (Figure 2B), although, the authors did not examine if the inclusions formed in this assay were capable of transmitting aggregation pathology to naïve cells. A more recent study has suggested that either addition of preformed TDP-43 amyloid-like fibrils or higher than typical TDP-43 levels in the cytoplasm can provoke the formation of cytoplasmic TDP-43 condensates independent of stress granules (Gasset-Rosa et al., 2019), suggesting that nucleation of TDP-43 aggregates may occur in the absence of typical membraneless-organelles. It remains to be established if the aggregates generated by phase transitions can seed the further aggregation of TDP-43.

### Intra- and Inter-cellular Prion-Like Spread of TDP-43 Aggregation

Overexpression of untagged or fluorescently-tagged TDP-43 in cultured mammalian cells can result in its mislocalization and aggregation in the cytoplasm (Johnson et al., 2009; Nonaka et al., 2009; Yamashita et al., 2009; Zhang et al., 2009, 2011; Barmada et al., 2010; Wang et al., 2010, 2013; Xu et al., 2010; Yang et al., 2010; Che et al., 2011, 2015; Furukawa et al., 2011; Gregory et al., 2012; Hong et al., 2012; Chang et al., 2013; Walker et al., 2013; Farrawell et al., 2015; Jiang et al., 2016; Tanaka and Hasegawa, 2016; Afroz et al., 2017; Zeineddine et al., 2017). Furthermore, disruption of the TDP-43 NLS leads to its accumulation and aggregation in the cytoplasm (a mutant denoted as TDP-43^ΔNLS^; Winton et al., 2008). Indeed, the first evidence for the seeded aggregation of TDP-43 in cells came from experiments utilizing an overexpression of TDP-43 with a C-terminal hemagglutinin (HA) tag (Furukawa et al., 2011). Furukawa et al. (2011) generated recombinant full-length TDP-43 and induced its fibrillar aggregation by vigorous agitation and subsequently introduced these fibrils to cells using lipofection. They found that introduction of TDP-43 aggregates to cells induced the aggregation of the HA-tagged TDP-43 in the cell cytoplasm, and that these cellular aggregates were polyubiquitinated and sarkosyl insoluble (Furukawa et al., 2011). The first evidence of the intercellular transmission of TDP-43 aggregation and relevance to patient pathology came when Nonaka et al. (2013) induced the aggregation of overexpressed HA-tagged TDP-43 in a neuronal cell line through lipofection of the insoluble protein fraction of human sALS or TDP-43 positive FTD patient brain samples. They observed that addition of either the FTD or sALS samples to cells resulted in the formation of HA-tagged positive TDP-43 positive inclusions. Additionally, they determined that the inclusions formed by the brain fractions in cultured cells could be used to seed further aggregation in cultures of naïve cells (Nonaka et al., 2013), providing substantial evidence supporting a pathological prion-like propagation of TDP-43 in FTD and ALS. Later studies showed similar results for the cell-to-cell spread of aggregation using stable cultures (Porta et al., 2018; Laferrière et al., 2019) overexpression cultures (Smethurst et al., 2016), co-cultures (Zeineddine et al., 2017), and even showed the axonal uptake and transport of TDP-43 seeds (Feiler et al., 2015).

Currently, cell cultured-based experiments provide the most well-established examinations of pathologic TDP-43 prion-like strains. Sequential deletion of amino acid segments, 20 residues in size, from the C-terminal region of TDP-43 highlighted amino acids 274–313 or 314–353 as being important for the cellular aggregation of TDP-43 (Shimonaka et al., 2016), suggesting that separate regions of the protein could be responsible for aggregation. Indeed, injection of amyloid fibrils composed of synthetic peptides composed of residues 274–313 or 314–353 resulted in the template-directed aggregation of wild-type TDP-43 or TDP-43^ΔNLS^ indicating that more than one region of the C-terminal region of the protein was capable of seeding pathologic aggregation (Shimonaka et al., 2016), however, the authors of this study did not note differences in aggregation characteristics. Although this provides evidence of possible strain-like characteristics of TDP-43, synthetic peptides provide little relevance to the pathological structures that may exist in disease. More recently, strong evidence for the existence of TDP-43 strains in human FTD patients was provided by Laferrière et al. (2019) when they carefully processed human tissue from FTD patients and ALS patients to enrich for pathological TDP-43. Examination of the size of the enriched TDP-43 assemblies from patients using velocity sedimentation showed that disease subtypes had different sized assemblies present, and that material from different disease subtypes as capable of inducing different levels of toxicity in HEK cells stably expressing HA-tagged TDP-43 and primary mouse cortical neurons (Laferrière et al., 2019). Most importantly, the authors showed that the toxicity of the TDP-43 assemblies to cultured cells correlated with the severity of the disease determined in the donor patient. It will be interesting to see if the disease severity will correlate with an *in vivo* model of TDP-43 prion-like propagation in future work.

### *In vivo* Seeding and Spread of TDP-43 Aggregation

Evidence supporting the prion-like propagation of TDP-43 aggregation *in vivo* occurred only recently. Porta et al. (2018) screened the prion-like properties of FTD patient brain extracts using cultured cells. Samples that had seeding activity were directly injected into the brains of mice expressing human TDP-43 with a mutated NLS signal under a doxycycline controllable promoter in their forebrain neurons (Igaz et al., 2011; Alfieri et al., 2014). It was found that injection of FTD patient-derived TDP-43 enriched samples led to the formation of pathologically relevant TDP-43 inclusions in the recipient mice (Porta et al., 2018). Additionally, careful immunohistochemical analysis of the injected mice showed that pathology appeared to spread through connected neuroanatomical tracts in a time-dependent manner, and interestingly, that in some cases endogenous mouse TDP-43 was colocalized with human TDP-43 inclusions. The authors then went on to inject seeding-competent, human-derived FTD material into non-transgenic wild-type mice, finding that the endogenous mouse TDP-43 could be seeded also, but to a lesser extent comparative to the doxycycline-regulated human TDP-43 transgenic mice (Porta et al., 2018). It was also determined that mice expressing a C-terminal fragment under a doxycycline regulatable element had a lower efficiency of seeding after injection with the FTD patient-derived samples, suggesting that the N-terminal regions of TDP-43 play an important role in its self-recognition and prion-like aggregation. Although the study by Porta et al. (2018) provides strong evidence for the prion-like role of TDP-43 in FTD, the experimental transmissibility of ALS patient-derived TDP-43 aggregates remains to be established.

## Fused in Sarcoma and C9orf72

### Fused in Sarcoma

Other than TDP-43 or SOD1, FUS plays an active role in the etiology of ALS owing to its presence in inclusions in a small percentage of ALS patients (Ling et al., 2013), and it containing several ALS-associated mutations (Kwiatkowski et al., 2009; Vance et al., 2009). Pathologically, FUS mostly forms basophilic inclusion bodies and was first found to play a role in a form of FTD called basophilic inclusion body disease (Munoz et al., 2009). Previous immunohistochemical analysis of basophilic inclusions in aytpical ALS patient tissue identified key constituents of stress granules [poly(A)-binding protein, TIA-1, and ribosomal protein S6] but not markers of other biomolecular condensates such as processing bodies or ribonucleoprotein particles (Fujita et al., 2008). Importantly, this work showed for the first time that stress granules play an important role in ALS pathogenesis.

Similar to TDP-43, FUS is an RBP which plays crucial roles in RNA metabolism (Belly et al., 2005; Fujii and Takumi, 2005; Andersson et al., 2008), but is also important for the DNA-damage repair response (Wang et al., 2008, 2013). Structurally, FUS belongs to the ten-eleven translocation (TET) protein family which are characterized by their N-terminal QSGY-rich domains, conserved RRMs, RGG-rich regions, and a C-terminal zinc finger motif (Morohoshi et al., 1998; Iko et al., 2004). Importantly, the N-terminal QSGY-rich domain (residues 1–165) of FUS is predicted to be a prion-like domain (PrLD; King et al., 2012), suggesting that this domain plays a role in the pathological aggregation of FUS observed in patients. However, the majority of ALS-associated mutations identified in FUS are localized to the N-terminal RGG-rich region (residues 165–276) or a short stretch (residues 495–526) of the C-terminal RGG-rich region (residues 449–526) which contains the FUS NLS (Abel et al., 2013); only one ALS-associated mutation (G156E) has been identified in the PrLD.

Examination of purified FUS PrLD has shown that it can undergo a phase transition to form hydrogels composed of amyloid-like fibrils (Han et al., 2012; Kato et al., 2012; Kwon et al., 2013). Additionally, EGFP-tagged full-length FUS can form biomolecular condensates in which the G156E mutation can exacerbate a transition from liquid to fibrous solid (Patel et al., 2015). Interestingly, although the ability of purified FUS to form aggregates is established, evidence suggesting that these assemblies can seed the aggregation of soluble FUS protein has only recently been established (Nomura et al., 2014). Nomura et al. (2014) generated purified GST-tagged FUS wild-type and the G156E mutant, finding that the G156E mutant was more prone to form fibrils and that these fibrils were capable of seeding purified wild-type FUS. They then went on to transfect rat-hippocampal primary neurons with both G156E FUS and wild-type FUS constructs, finding that inclusions would only form for the mutant and that these inclusions would recruit the plasmid derived wild-type FUS (Nomura et al., 2014). Other than the work by Nomura et al. (2014), there has been little research carried out on examining the prion-like characteristics of FUS. Future work examining if FUS has bona-fide prion-like characteristics (seeding from exogenous aggregates, cell-to-cell propagation) will be important in determining if a prion-like mechanism is common across the main aggregation-prone proteins in ALS (SOD1, TDP-43, FUS).

### C9orf72

Hexanucleotide repeat (GGGGCC) expansions in *C9orf72* were first identified in DeJesus-Hernandez et al. (2011) and Renton et al. (2011) and are now thought to be responsible for approximately 40% of fALS cases (Majounie et al., 2012). Typically, the number of repeats in the healthy population is ≤11, whereas the number of repeats in an individual suffering from *C9orf72*-associated fALS can number in the thousands (van Blitterswijk et al., 2013; Suh et al., 2015). Although exact functions for the protein encoded by *C9orf72* is yet to be fully elucidated, there is evidence to suggest that it has a role as a guanine nucleotide exchange factor for small GTPases (Wu et al., 2011; Levine et al., 2013; Iyer et al., 2018). The protein encoded by *C9orf72* is also implicated in other cellular functions such as autophagy (Sellier et al., 2016; Webster et al., 2016) and vesicular transport (Aoki et al., 2017). Although there is little known about the function(s) of the C9orf72 protein, understanding of the mechanisms by which disease arises consequently from repeat expansions has proceeded rapidly.

**Figure 3:**
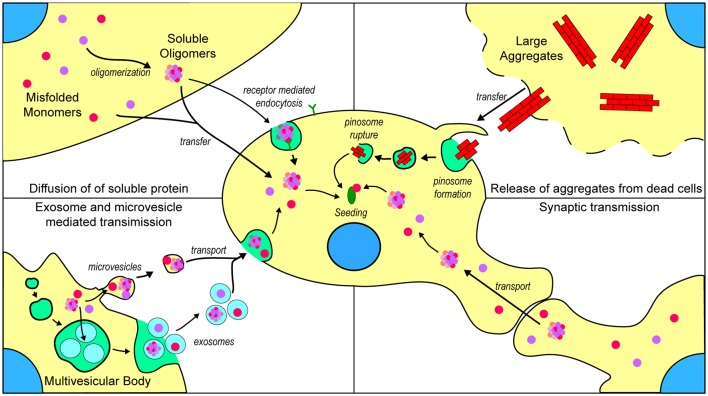
Mechanisms by which prion-like ALS-associated protein aggregates may transfer between cells. (Top left) Once formed, misfolded monomers and soluble oligomers can potentially exit the cell through diffusion and enter nearby cells either through diffusion or receptor-mediated endocytosis. (Top right) Cells that are dead or dying can potentially release larger insoluble aggregates that can be taken up into nearby naïve cells through micropinocytosis. (Bottom left) Misfolded and oligomeric proteins are capable of being loaded into either microvesicles or exosomes for transport to nearby cells. (Bottom right) Misfolded monomers or soluble oligomers are capable of being transported across axon terminals to naïve cells.

There are now thought to be several mechanisms by which repeat expansions in *C9orf72* cause toxicity, including a loss of function, transcription into long repetitive RNA that forms foci composed of sense or antisense RNA (Donnelly et al., 2013; Gendron et al., 2013; Lagier-Tourenne et al., 2013; Mizielinska et al., 2013; Zu et al., 2013), and the translation of repeat RNA into five different dipeptide repeat (DPR) proteins (poly-GR, poly-GP, poly-GA, poly-PR, and poly-PA; Mori et al., 2013). Currently, there is little evidence to suggest a prion-like propagation of any of the DPRs generated in C9orf72-associated ALS. One study by Westergard et al. (2016) used a battery of cell culture experiments to examine DPR spread between cells. They determined that all DPRs, with the exception of poly-PR, were capable of transfer between cells through anterograde and retrograde mechanisms, and that this transfer can occur through both exosome-dependent and exosome-independent mechanisms Modes of transfer of prion-like material is summarized in Figure 3. Although this work provides strong evidence for the cell-to-cell transfer of certain DPRs, it is still not established if DPRs can assume a conformation with prion-like characteristics or if they can seed further aggregation of other DPRs.

## Perspective and Conclusion

It is now established that prion-like mechanisms play key roles in the cell-specific nature and cell-to-cell spread of pathology in neurodegenerative disease (Prusiner, 2013). In particular, ALS shows the hallmarks of prion-like propagation owing to the spread of both symptoms and pathology throughout affected tissues (evidence is summarized in Table 1). Different types of research including biophysical, biochemical, and biomedical studies have increased our understanding of the contribution of prion-like mechanisms to ALS. Cell-free *in vitro* examination of the aggregation and seeding characteristics of prion-like proteins associate with ALS have provided useful information on the kinetics of aggregation and the biophysical traits of the substrate and seed species. Cell culture experiments have helped establish the cellular mechanisms by which proteinaceous aggregation may occur, and how cells may facilitate the transmission of the prion-like particles. Research using animal models has been effective at establishing the biochemical and phenotypic profiles of some prion-like proteins and strains in ALS and has also helped in determining if material from patients suffering ALS or FTD have prion-like (PrP-like) properties (Ayers et al., 2014, 2016a,b; Bergh et al., 2015; Lang et al., 2015; Bidhendi et al., 2016, 2018; Laferrière et al., 2019).

Although great progress has been made in the understanding of the prion-like nature of ALS, what remains to be elucidated are high-resolution structural models of specific ALS-associated prion strains that are related to disease pathology. While recent work has identified several fibrillar polymorphs of the TDP-43 low complexity domain (Cao et al., 2019), it remains to be established if these conformational assemblies have relevance to pathogenicity *in vivo*, missing a crucial link between conformation of the amyloid-like assemblies and their toxicity. If we are to develop effective therapeutics against such polymorphic protein assemblies, we need to know what to target. Future experiments investigating the roles of prion-like propagation in human disease should combine *in vivo* models of seeding and neuroanatomical spread, cell culture studies of seeding potency of different strains, and the structural resolving power of cryo-electron microscopy for structure determination.

The existence of prion-like conformational strains in ALS provides not only a challenging hurdle to overcome experimentally and therapeutically, but also provides opportunities to develop effective therapies. With access to disease-specific structural models of prion-like aggregates, researchers have the capability to perform large-scale structure-based small molecule docking screens, which may identify compounds that could alleviate or prevent the occurrence or spread of pathology in prion-like diseases (Jiang et al., 2013). Furthermore, structure-based design of monoclonal antibodies targeted against misfolding specific epitopes is a growing space with great potential. Given a prion strain structure, an antibody could be rationally designed to target a specific conformation of protein assembly to prevent further spread or promote clearance (Peng et al., 2018; Silverman et al., 2018). An advantage of this approach is that the use of monoclonal antibodies would overcome target distraction through their high binding specificity.

Lastly, the role of prion-like propagation of pathologic proteins in ALS is likely tied to the mostly idiopathic nature of the disease. Whilst there are mutations that are ALS-causative, the majority of cases remain sporadic, suggesting that an environmental trigger may exist. From this, a question that must be asked is whether or not all, or a set, of sporadic cases of ALS are the result of a still-unidentified prion-like agent in the environment. Recent research has provided evidence supporting the Braak hypothesis (Braak et al., 2003) that pathologically aggregated α-synuclein can propagate from the gut, through the vagus nerve, to the ventral midbrain (Kim et al., 2019). Furthermore, some patients who received human growth hormone from patients suffering from Creutzfeld-Jakob disease (CJD) developed CJD themselves (Gibbs et al., 1985). Surprisingly, some of the patients who received growth hormone from CJD sufferers were found to have amyloid-β pathology, suggesting that amyloid-β had also propagated in these cases (Jaunmuktane et al., 2015). Considering the above evidence, there is a possibility for similar mechanisms to be occurring in ALS, however, there is currently no strong evidence supporting this hypothesis. Epidemiological studies may provide further insight into links between the populations that develop sporadic ALS beyond just genetic background.

## Author Contributions

LM wrote the initial manuscript. SP edited and revised the manuscript. JY edited and revised the manuscript. NC conceived, edited and revised the manuscript.

## Conflict of Interest

The authors declare that the research was conducted in the absence of any commercial or financial relationships that could be construed as a potential conflict of interest.
