# Corrigendum: Prion-Like Propagation of Protein Misfolding and Aggregation in Amyotrophic Lateral Sclerosis

**DOI:** 10.3389/fnmol.2019.00311

**Published:** 2020-01-21

**Authors:** Luke McAlary, Steven S. Plotkin, Justin J. Yerbury, Neil R. Cashman

**Affiliations:** ^1^Illawarra Health and Medical Research Institute, University of Wollongong, Wollongong, NSW, Australia; ^2^Molecular Horizons and School of Chemistry and Molecular Bioscience, Faculty of Science, Medicine and Health, University of Wollongong, Wollongong, NSW, Australia; ^3^Department of Physics and Astronomy, University of British Columbia, Vancouver, BC, Canada; ^4^Genome Sciences and Technology Program, University of British Columbia, Vancouver, BC, Canada; ^5^Djavad Mowafaghian Centre for Brain Health, University of British Columbia, Vancouver, BC, Canada

**Keywords:** amyotrophic lateral sclerosis, protein misfolding, protein aggregation, prion, proteostasis

In the original article, there was a mistake in Table 1 as published. The incorrect original research articles were referenced for some of the table cells. The corrected Table 1 appears below.

**Table 1:** Evidence supporting the prion-like characteristics of ALS-associated proteins.

**Protein/Gene**	***in vitro* fibril formation**	***in vitro* fibril seeding**	**Cell seeding with *in vitro* protein**	**Cell-to-cell propagation**	**Human to cell propagation**	***in vitro* to animal propagation**	**Animal to animal propagation**	**Human to animal propagation**
SOD1	✓ Chattopadhyay et al. (2008)	✓ Chia et al. (2010)	✓ Munch et al. (2011)	✓ Grad et al. (2014)	✓ Pokrishevsky et al. (2017)	✓ Ayers et al. (2016a)	✓ Ayers et al. (2014)	✓ Ekhtiari Bidhendi et al. (2018)
TDP-43	✓ Johnson et al. (2009)	✓ Furukawa et al. (2011)	✓ Furukawa et al. (2011)	✓ Nonaka et al. (2013)	✓ Nonaka et al. (2013)	–	–	✓ Porta et al. (2018)
FUS	✓ Nomura et al. (2014)	✓ Nomura et al. (2014)	–	–	–	–	–	–
C9orf72 (DPRs)	–	–	–	✓ Westergard et al. (2016)	–	–	–	–

The authors apologize for this error and state that this does not change the scientific conclusions of the article in any way. The original article has been updated.
